# Double‐Hydrophobic‐Coating through Quenching for Hydrogels with Strong Resistance to Both Drying and Swelling

**DOI:** 10.1002/advs.201903145

**Published:** 2020-01-24

**Authors:** Md. Tariful Islam Mredha, Hong Hieu Le, Jiaxi Cui, Insu Jeon

**Affiliations:** ^1^ School of Mechanical Engineering Chonnam National University 77 Yongbong‐ro, Buk‐gu Gwangju 61186 Republic of Korea; ^2^ INM ‐ Leibniz Institute for New Materials Campus D2 2 Saarbrücken 66123 Germany; ^3^ Institute of Fundamental and Frontier Sciences University of Electronic Science and Technology of China Chengdu Sichuan 610054 China

**Keywords:** double‐hydrophobic‐coating, drying resistance, hydrogels, quenching, swelling resistance

## Abstract

In recent years, various hydrogels with a wide range of functionalities have been developed. However, owing to the two major drawbacks of hydrogels—air‐drying and water‐swelling—hydrogels developed thus far have yet to achieve most of their potential applications. Herein, a bioinspired, facile, and versatile method for fabricating hydrogels with high stability in both air and water is reported. This method includes the creation of a bioinspired homogeneous fusion layer of a hydrophobic polymer and oil in the outermost surface layer of the hydrogel via a double‐hydrophobic‐coating produced through quenching. As a proof‐of‐concept, this method is applied to a polyacrylamide hydrogel without compromising its mechanical properties. The coated hydrogel exhibits strong resistance to both drying in air and swelling in multiple aqueous environments. Furthermore, the versatility of this method is demonstrated using different types of hydrogels and oils. Because this method is easy to apply and is not dependent on hydrogel surface chemistry, it can significantly broaden the scope of next‐generation hydrogels for real‐world applications in both wet and dry environments.

## Graphical abstract

A double‐hydrophobic‐coating method using a hydrophobic polymer and a viscous oil prepared via quenching is developed to fabricate hydrogels with strong resistance to drying in air and swelling in multiple aqueous environments. The method is not dependent on the type of oil or the surface chemistry of the hydrogel used; therefore, it is applicable to a wide range of hydrogels.

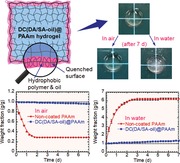

Hydrogels are ecofriendly materials with a high water content, in which hydrophilic polymer networks are highly solvated by water to produce a 3D tissue‐like structure.^1^, ^2^, ^3^ They have attracted considerable attention in recent years owing to their extraordinary biomimetic properties (e.g., they are biocompatible, soft, flexible, and permeable to various biomolecules, drugs, minerals, and ions).^4^, ^5^, ^6^ Furthermore, research efforts to reduce the use of fossil fuels by replacing plastic materials with water‐based hydrogel materials is ongoing.^7^ In the last decade, numerous hydrogels with good mechanical, structural, biological, and electrical properties have been developed.^1^, ^2^, ^3^, ^4^, ^5^, ^6^, ^7^, ^8^, ^9^, ^10^, ^11^ Hierarchical,^2^, ^9^ hydrophobic/hydrophilic,^3^ alginate/polyacrylamide,^4^ double‐network,^11^ and polyampholyte^12^ hydrogels are some examples of recently designed advanced hydrogels with extraordinary properties. Unfortunately, because hydrogels contain a large amount of water, almost all hydrogels inevitably dry out in air, reducing their flexibility and functionality.^9^, ^13^, ^14^ For example, a 30 × 10 × 1.5 mm^3^ Ca‐alginate hydrogel dries in air within 5 h.^9^ Additionally, most hydrogels swell significantly when immersed in water due to the hydrophilic nature of the polymers, which can decrease their mechanical properties dramatically.^15^ For example, a typical as‐prepared polyacrylamide (PAAm) hydrogel swells to four times its original volume in 4 h when immersed in water.^16^

Until now, only a few hydrogels have been reported that exhibit good resistance to drying or swelling.^3^, ^15^, ^17^, ^18^, ^19^, ^20^ However, none of the hydrogels developed thus far have shown stability in both air and water, which limits their practical applications.^13^ Since drying and swelling are among the inherent characteristics of hydrogels, it is a significant challenge to design hydrogels with strong resistance to both drying and swelling. To broaden the range of hydrogel applications, it is imperative to develop a suitable method for overcoming both these major drawbacks.

Hydrogels with drying resistance were originally developed by incorporating high‐density hygroscopic salts in the bulk hydrogel system^17^ or by encapsulating the hydrogels within an elastomeric material.^18^, ^19^ However, the former may produce a toxic effect owing to the presence of high‐density salts, while the latter makes hydrogels impermeable to many important chemicals, such as ions and drug molecules, eliminating an important function of hydrogels. Complicated microchanneling in the elastomeric layer was therefore necessary to induce diffusion and promote reactions of different species through the elastomeric surface.^18^ Furthermore, covering the complex 3D shapes of hydrogels with a predesigned elastomeric film is challenging. None of the methods reported thus far have demonstrated long‐term efficiency to prevent air‐drying. On the other hand, hydrogels with swelling resistance are usually designed by modulating the internal chemical structure of the hydrogel. For example, Sakai et al.^15^ developed swelling‐resistant hydrogels using hydrophilic and thermoresponsive polymers, in which two independently occurring effects (swelling and shrinking) of the two different polymer networks oppose each other. Such hydrogels are also developed by adjusting the composition of hydrophobic and hydrophilic components in the hydrogel matrix.^3^, ^20^ These design strategies are ultimately limited to particular classes of chemicals and are therefore restricted in applicability.

Mammalian skin forms a barrier with selective permeability; it limits the diffusion of water and electrolytes while allowing the absorption of respiratory gases and transdermal drugs when necessary.^21^, ^22^ Skin is a tough multihydrophobic composite of polymers (e.g., cells and proteins) and oily substances (e.g., epidermal and sebaceous lipids), where cells/proteins provide mechanical integrity and lipids cover the intercellular space to regulate the essential permeability barrier of the skin.^21^ Inspired by the structure of skin, we devised a polymer and oil‐based double‐hydrophobic‐coating method for constructing a robust permeability barrier on the hydrogel surface without modifying its bulk structure, to selectively prevent both outward and inward diffusion of water (**Figure** **1**). This method includes three simple steps. In the first step, a long‐chain hydrophobic monomer is polymerized by a pretrapped radical initiator on the outermost layer of the hydrogel to produce a hybrid structure comprising hydrophobic polymers with hydrophilic polymers at the hydrogel surface (Figure 1I,II). We used 2,2‐azobisisobutyronitrile (AIBN), a commonly used initiator for the polymerization of hydrophobic monomers, as the radical initiator. After the fabrication of the original hydrogel, AIBN was physically entrapped on the surface of the gel as follows. The hydrogel was immersed in an AIBN/benzene solution for a certain period of time, allowing the AIBN/benzene molecules to physically adsorb onto the surface layer of hydrogel. The AIBN/benzene molecules cannot penetrate far inside the hydrogel as they are nonmiscible with water. Then, the low‐boiling‐point benzene was evaporated from the surface by heating the gel at 120 °C for ≈2 min, leaving only AIBN deposited physically onto the surface layer of the hydrogel. Subsequently, the gel was immersed into a hydrophobic monomer bath at 120 °C (Figure 1I). The surface AIBN molecules then initiate polymerization, leading to outward growth of a hydrophobic polymer from the surface layer of the hydrogel (Figure 1II). In this process, the hydrophobic monomer does not penetrate far into the bulk of the hydrogel owing to its hydrophobicity, nonmiscibility with water, and rapid polymerization. The growth of the polymer and the thickness of the coated layer can be tuned by adjusting the polymerization time. In the second step, an oil treatment was applied by immersing the gel in a hot silicone oil bath (120 °C) for 15 min (Figure 1III). In this process, hot oil was readily absorbed by the outermost hydrophobic polymer layer of the hydrogel while simultaneously washing away any unreacted monomer. In the third step, the hydrogel was removed from the hot silicone oil bath and quenched quickly by immersing it in a room temperature silicone oil bath (25 °C) (Figure 1IV). Consequently, the expanded surface of the coated hydrogel shrank and locked the oil in the polymer layer, producing a permanent shield on the hydrogel surface. This double‐hydrophobic‐coated shield was capable of simultaneously preventing water molecules from evaporating out of the hydrogel in air or entering it in water, making the hydrogel strongly resistant to both drying and swelling.

**Figure 1:**
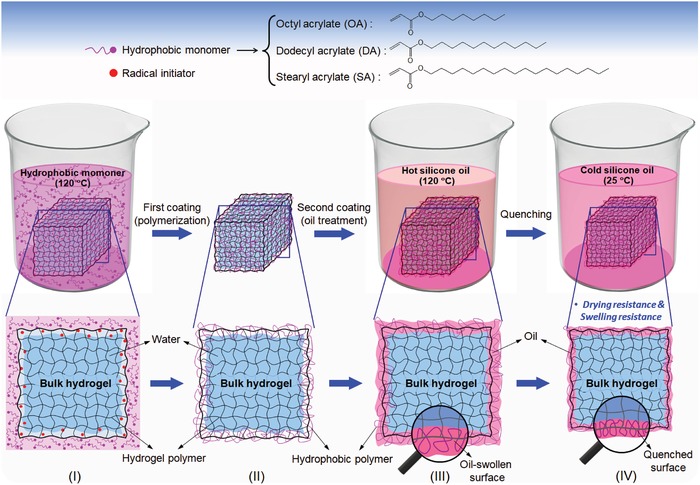
Design strategy for fabricating hydrogels with strong resistance to both drying and swelling. First, a hydrophobic polymer layer was created on the surface of the hydrogel (I, II). The gel was then immersed in hot silicone oil, where the hydrophobic polymer absorbed oil easily at high temperature (120 °C) (III). Subsequently, the gel was immersed in a silicone oil bath at room temperature (25 °C), quenching the surface to produce a robust homogeneous structure of hydrophobic polymer and oil (IV), which provides a strong barrier to the transfer of water and significantly reduces drying and swelling.

To form the double‐hydrophobic‐coating, we used acrylate‐based hydrophobic monomers with three different chain lengths: octyl acrylate (OA; short chain), dodecyl acrylate (DA; medium‐length chain), and stearyl acrylate (SA; long chain) (Figure 1). Unless otherwise noted, the polymerization of the hydrophobic monomers was carried for ≈30 min at 120 °C. Silicone oil with a viscosity of 1000 cSt (at 25 °C) was used as the oil, unless otherwise specified. The method was initially applied to typical PAAm hydrogels. Double‐hydrophobic‐coated PAAm hydrogels fabricated using OA, DA, SA, and a DA/SA mixture (1:1 by volume) are denoted as DC(OA‐oil)@PAAm, DC(DA‐oil)@PAAm, DC(SA‐oil)@PAAm, and DC(DA/SA‐oil)@PAAm, respectively. The detailed fabrication processes are described in the Supporting Information.

**Figure** **2**a shows that the double‐hydrophobic‐coated PAAm hydrogel exhibited no noticeable change in size when left in air for 7 d at ≈25 °C, whereas its noncoated counterpart shrank considerably and dried completely within 2 d. We quantitatively determined the change in the weight of the gel in air up to 7 d (Figure 2b). The results indicated that the DC(DA/SA‐oil)@PAAm hydrogel lost only 4.1 ± 0.07 wt% after 7 d, whereas the noncoated PAAm gel lost all its water content (71.2 ± 0.3 wt%). Single‐hydrophobic‐coated gels prepared using either only the oil (SC(oil)@PAAm) or the hydrophobic polymer (SC(DA/SA)@PAAm) lost 71.5 ± 0.2 and 35 ± 2.5 wt%, respectively. These results reveal the synergistic effect of the hydrophobic polymer and oil in the double‐hydrophobic‐coating. The continuous oil medium within the hydrophobic polymer layer produced a homogeneous shield on the hydrogel surface to prevent air‐drying, which was impossible to achieve with any single hydrophobic component (either hydrophobic polymer or oil). We quantitatively measured the coating density; an average density of ≈0.024 g cm^−2^ at the hydrogel surface was achieved for the best‐performing double‐hydrophobic‐coated gel (DC(DA/SA‐oil)@PAAm).

**Figure 2:**
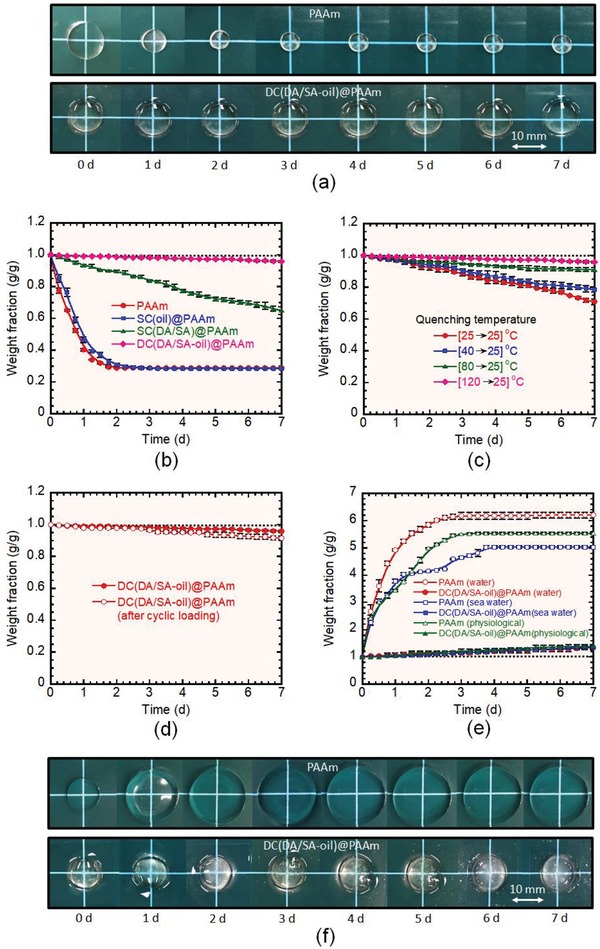
Evaluation of the drying and swelling properties under multiple conditions. a) Photographs of PAAm and DC(DA/SA‐oil)@PAAm hydrogels in air over 7 d. b) Comparison of the air‐drying properties of the noncoated (PAAm), single‐coated hydrogels (SC(oil)@PAAm and SC(DA/SA)@PAAm), and double‐coated hydrogels (DC(DA/SA‐oil)@PAAm). c) Air‐drying properties of the DC(DA/SA‐oil)@PAAm hydrogel fabricated at different quenching temperatures. d) Air‐drying properties of the DC(DA/SA‐oil)@PAAm hydrogel before and after ten consecutive loading–unloading cycles at a compressive strain of 0.5. e) Swelling properties of noncoated PAAm and DC(DA/SA‐oil)@PAAm hydrogels in water (25 °C), seawater (25 °C), and physiological conditions (0.16 m NaCl, 37 °C). f) Photographs of PAAm and DC(DA/SA‐oil)@PAAm hydrogels while underwater for 7 d.

The chain length of the hydrophobic polymer affected the air‐drying performance of the hydrogel. The medium‐length hydrophobic monomer (DA) exhibited better efficiency than the shorter one (OA). However, the longest monomer (SA) exhibited unexpectedly poor performance (Figure S1, Supporting Information); this is because the glass transition temperature (*T*_g_) of the SA homopolymer (≈48 °C) is higher than the ambient temperature (≈25 °C),^23^ causing it to create an inhomogeneous hard structure on the hydrogel surface under ambient conditions. Upon combining DA and SA (1:1 by volume) for copolymerization, inhomogeneity was not observed, likely owing to a decrease in the *T*_g_ of the copolymer because the *T*_g_ of the DA homopolymer is very low (−55 °C).^24^ DA/SA (1:1) therefore provided good hydrophobicity, while remaining homogeneous and exhibiting the best air‐drying performance.

Oil viscosity is another important parameter for tuning the air‐drying performance. Coatings based on high‐viscosity oils (1000 and 2000 cSt) provided far better drying resistance than a low‐viscosity oil (100 cSt) coating (Figure S2, Supporting Information). This is comparable to human skin, which consists of high‐viscosity fats. The polymer chain length and molecular weight of high‐viscosity silicone oil are higher than that of low‐viscosity oil.^25^ The longer polymer chain of the high‐viscosity oil probably produces a more stable entangled structure with the hydrophobic polymer in the coating region, which results better air‐drying performance. Hereafter, the best‐performing DC(DA/SA‐oil)@PAAm hydrogel prepared with 1000‐cSt silicone oil was used for further study, unless otherwise noted.

The quenching process had a remarkable effect on the properties of the double‐hydrophobic‐coating; hence, it affected the air‐drying performance of the hydrogel (Figure 2c). When a sufficiently high temperature (120 °C) is used for treatment of the hydrophobic polymer and oil‐coated hydrogel, followed by rapid quenching at a low temperature (25 °C), sudden shrinkage of the surface structure occurs, which can trap oil within the hydrophobic polymer layer and produce a robust polymer–oil homogeneous surface. This phenomenon cannot be realized if the treatment temperature is too low. To demonstrate this, we quantitatively determined the amount of oil absorbed by the coating after treatment at 40 and 120 °C followed by room temperature (25 °C) quenching. The double‐hydrophobic‐coated hydrogel treated at a high temperature (120 °C) absorbed ≈36 wt% oil (of the total amount of hydrophobic polymer and oil); while the gel treated at a low temperature (40 °C) absorbed only ≈11 wt% oil. Therefore, the double‐hydrophobic‐coated hydrogel treated at a high temperature (120 °C) exhibited significantly better drying resistance than those treated at lower temperatures (25, 40, and 80 °C).

We evaluated the mechanical properties of the double‐hydrophobic‐coated hydrogels via both tensile and compression testing (Figures S3 and S4, Supporting Information) and discovered that the mechanical performance of the hydrogels did not decrease after the coating process; rather, the coated hydrogel exhibited better tensile properties than the noncoated one, probably due to an increase in the polymer density from the coating process (≈15% volume shrinkage). Interestingly, the coating layer of the hydrogels was not affected by harsh mechanical treatments. For example, the air‐drying performance of the DC(DA/SA‐oil)@PAAm hydrogel after ten consecutive loading–unloading cycles (at 50% compressive strain) was close to the performance of the intact coated hydrogel without mechanical treatment (Figure 2d). Additionally, the two gels exhibited similar loading–unloading pathways (Figure S5, Supporting Information). This strongly suggests that our method produced a robust coating structure and interface on the hydrogel surface that can survive in harsh mechanical environments.

In addition to preventing the outward transfer of water, the double‐hydrophobic‐coated layer can prevent the inward transfer of water. We immersed the DC(DA/SA‐oil)@PAAm hydrogel (after 7 d drying in air) in multiple aqueous environments (i.e., pure water, seawater, and a physiologically relevant saline solution containing 0.16 m NaCl (Figure 2e)). While the noncoated PAAm hydrogel swelled rapidly and significantly, the coated DC(DA/SA‐oil)@PAAm hydrogel exhibited strong resistance to swelling (Figure 2f). No apparent volume change of the coated gel was observed, even after prolonged immersion in water. After 7 d, the swelling ratio (the ratio of diameter before and after immersion) in water was ≈1.06 (close to the ideal nonswelling value of 1.0); in contrast, the swelling ratio of the noncoated gel was as large as ≈1.90. After 7 d immersion in water (25 °C), sea water (25 °C), and saline solution (37 °C), the weight of the DC(DA/SA‐oil)@PAAm hydrogel increased by only 33 ± 7, 34 ± 4, and 38 ± 6 wt%, respectively, whereas the weight of the noncoated PAAm gel increased by 520 ± 11, 403 ± 5, and 453 ± 4 wt %, respectively. Previously, swelling‐resistant hydrogels were developed by engineering the internal chemical structure; our method does not alter the chemical structure and retains the properties of the original hydrogel formulation.^3^, ^15^, ^20^ The double‐hydrophobic‐coated layer on the surface can prevent the penetration of water, similar to mammalian skin.

Both the surface and internal structure of the hydrogel were characterized via scanning electron microscopy (SEM) using an air‐dried sample (xerogel). While the surface of the noncoated PAAm gel was flat (Figure S6, Supporting Information), the coated gel surface had a compact and homogeneously patterned texture (**Figure** **3**a and Figure S7a, Supporting Information). The coated region can clearly be identified in the cross‐sectional view (Figure 3b and Figure S7b, Supporting Information), in which the hydrophobic polymer appears to be bonded tightly with the bulk gel network. No defects (unbonded regions) were observed at the interface. The thickness of the coated layer was uniform and slightly increased after oil treatment with quenching, reaching a final thickness of ≈200 µm (Figure 3b). The thickness of the coated layer, which connected to the density of the coating, can be adjusted by controlling the polymerization time. With a very short polymerization time (≈15 min), a nonuniform coated layer with a thickness of ≈100 µm was achieved, while with a long polymerization time (≈45 min), the coated layer grew as thick as ≈350 µm (Figure S8, Supporting Information). As expected, the coating thickness affected the drying resistance; the gel obtained by ≈15 min polymerization exhibited poor air‐drying performance in comparison to those obtained by ≈30 and ≈45 min polymerization (Figure S8d, Supporting Information). We found that ≈30 min polymerization was enough to achieve a stable uniform coated layer with a thickness of ≈200 µm and a coating density of ≈0.024 g cm^−2^, which exhibited best air‐drying performance.

**Figure 3:**
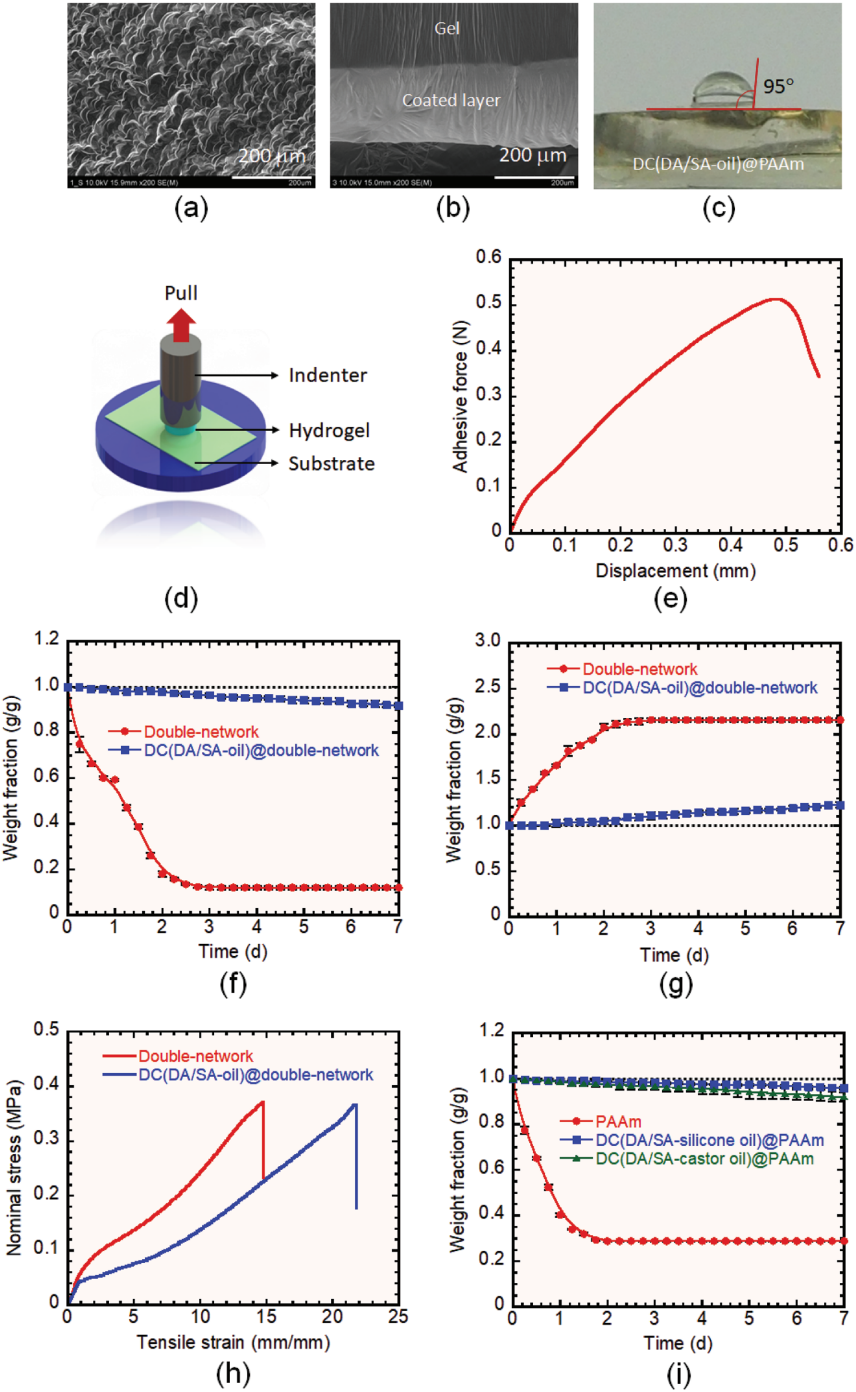
SEM images of DC(DA/SA‐oil)@PAAm hydrogel showing a) surface and b) cross‐sectional structures. c) Water contact angle measurement on the DC(DA/SA‐oil)@PAAm hydrogel surface. d) Experimental setup of the tack test for evaluation of surface adhesion. e) Adhesive force as a function of displacement for the DC(DA/SA‐oil)@PAAm hydrogel, obtained via the tack test. Comparison of f) air‐drying, g) water‐swelling, and h) tensile properties of the noncoated and double‐coated Ca‐alginate/PAAm double‐network hydrogels. i) Comparison of the air‐drying properties of noncoated and double‐coated PAAm hydrogels fabricated using silicone oil and castor oil.

The single hydrophobic polymer coating process significantly increased the water contact angle on the hydrogel surface. The noncoated PAAm hydrogel had a contact angle of 30° (Figure S9, Supporting Information), whereas the SC(DA/SA)@PAAm hydrogel had a contact angle of 111° (Figure S10, Supporting Information). These results indicate that the hydrophobic polymer network covered the hydrophilic surface of the hydrogel efficiently. After the second coating via oil treatment with quenching, the contact angle decreased slightly to 95°, because the surface roughness reduced owing to the oil treatment. However, this is still notably larger than the contact angle of the noncoated PAAm hydrogel (Figure 3c).

The double‐hydrophobic‐coating on the hydrogel surface serendipitously produced an adhesive surface. The adhesive strength and energy were evaluated by a standard tack test using a glass substrate (Figure 3d).^26^ The DC(DA/SA‐oil)@PAAm hydrogel exhibited an impressive adhesive strength of 6.4 ± 0.4 kPa (0.5 ± 0.03 N) and a debonding energy of 2.2 ± 0.2 J m^−2^ (Figure 3e). This adhesion likely originates from the physical adsorption process of the free long alkyl chain of the hydrophobic polymer on the hydrogel surface. This kind of physical adsorption‐based adhesion may also be applicable for various dry surfaces (e.g., metals, ceramics, and plastics) and wet surfaces (e.g., hydrogels and biotissues); however, future extensive studies should be carried out to further clarify this. The adhesive property of our system is comparable to commonly used mussel‐inspired catechol‐based hydrogels and other recently developed adhesive hydrogels that rely on supramolecular interactions for adhesion (Table S1, Supporting Information).^26^, ^27^, ^28^, ^29^, ^30^, ^31^ Notably, while most adhesive hydrogels (apart from nanoparticle adhesives) require internal modification of the chemical structure—which ultimately changes the properties compared to the original unmodified hydrogel—our strategy only alters the surface structure. Hence, this approach could be used to easily turn various nonadhesive hydrogels into adhesive hydrogels, while retaining the original bulk properties. Because our method is based on the physical entanglement of a hydrophobic polymer on the surface of a hydrogel, it is applicable to a wide range of network hydrogels, regardless of their surface chemistry.

To investigate this further, we applied our double‐hydrophobic‐coating method to a Ca‐alginate/PAAm double‐network hydrogel, which is a widely used tough and stretchable hydrogel.^4^, ^32^ Although the hydrogel contained ≈90 wt% water, the coating was capable of maintaining the water content in air for a prolonged period. Figure 3f shows that although the noncoated double‐network hydrogel dried completely within 2 d, the DC(DA/SA‐oil)@double‐network hydrogel dried very slowly and exhibited a reduction of only 8.1 ± 0.9 wt% after exposure to air for 7 d. The method was equally effective for preventing the swelling of this hydrogel. The noncoated hydrogel swelled significantly and rapidly, reaching its swelling equilibrium by increasing 116 ± 2.1 wt% (relative to its original weight) in 2 d, whereas the DC(DA/SA‐oil)@double‐network hydrogel swelled very slowly during the initial 2 d, increasing by only 22.6 ± 3.3 wt% (relative to its original weight) after immersion for 7 d in water (at 25 °C) (Figure 3g). These results clearly indicate that our double‐hydrophobic‐coating method is effective for preventing the transfer of water in or out of various hydrogels.

We also evaluated the mechanical properties of the Ca‐alginate/PAAm double‐network hydrogel. The coated and noncoated hydrogels exhibited similar tensile (Figure 3h and Figure S11, Supporting Information) and compression (Figure S12, Supporting Information) properties, indicating that the proposed method did not significantly affect the internal chemical structure of the hydrogel. The tough DC(DA/SA‐oil)@double‐network hydrogel had an average tensile modulus, strength, and strain of 0.05 ± 0.004 MPa, 0.4 ± 0.06 MPa, and 21 ± 2 mm mm^−1^, respectively, and an average compressive modulus and strength of 0.16 ± 0.02 and 3.3 ± 0.2 MPa (at a compressive strain of 0.9), respectively. In conjunction with its strong resistance to both drying and swelling, this hydrogel is likely to find numerous applications in both air and wet environments.

Interestingly, the performance of the double‐hydrophobic‐coating was independent of the type of oil used. We evaluated the efficacy of our method using castor oil (610 cSt at 25 °C), which is an edible and biocompatible vegetable oil used for many healthcare purposes.^33^ The DC(DA/SA‐castor oil)@PAAm hydrogel performed similarly to a double‐coated hydrogel fabricated using silicone oil (1000 cSt at 25 °C) (DC(DA/SA‐silicone oil)@PAAm) (Figure 3i). This suggests that the edible oil‐coated hydrogels prepared using the proposed method can be applied to living systems, in cases where potential toxicity from the type of oil might be a concern. However, future extensive biological assessment should be carried out to confirm this unambiguously.

In summary, we proposed a bioinspired, versatile, and noninvasive method for fabricating hydrogels with strong resistance to both drying and swelling. The method is based on double‐hydrophobic‐coating of a hydrophobic polymer (containing long alkyl chains) and viscous oil through quenching, which produces a ≈200 µm thick skin‐like robust hybrid layer on the hydrogel surface (without compromising its internal chemical structure or properties), which prevents both outward and inward transfer of water related to drying and swelling. The fabricated double‐hydrophobic‐coated hydrogels exhibited excellent stability in air and multiple aqueous environments (water, seawater, and physiological saline solution). Additionally, the surface of the hydrogels serendipitously adhered to solid substrates owing to the adsorption of long alkyl chains of the hydrophobic polymer. This physical method is independent of the surface chemistry of hydrogels and the type of oil used. Therefore, our method overcomes the major drawbacks (drying and swelling) associated with hydrogels while paving the way for numerous applications of this elite class of materials in diverse air, water, marine, and physiological environments.

## Experimental Section

Experimental details are provided in the Supporting Information.

## Conflict of Interest

The authors declare no conflict of interest.

## Supporting information

Supporting Information
