# Supplementary material for: Double‐Hydrophobic‐Coating through Quenching for Hydrogels with Strong Resistance to Both Drying and Swelling
Source: Adv Sci (Weinh). 2020 Jan 24;7(6):1903145. doi: 10.1002/advs.201903145 (PMC7080539; doi:10.1002/advs.201903145)
Supplement: Supplementary file 1 — Supporting Information [file ADVS-7-1903145-s001.pdf]

Copyright WILEY-VCH Verlag GmbH & Co. KGaA, 69469 Weinheim, Germany, 2020.

ADVANCED SCIENCE
Open Access

Supporting Information

for *Adv. Sci.,* DOI: 10.1002/advs.201903145

Double-Hydrophobic-Coating through Quenching for Hydrogels with Strong Resistance to Both Drying and Swelling

*Md. Tariful Islam Mredha, Hong Hieu Le, Jiaxi Cui, and Insu Jeon**

Copyright WILEY-VCH Verlag GmbH & Co. KGaA, 69469 Weinheim, Germany, 2018.

Supporting Information

Double-hydrophobic-coating through quenching for hydrogels with strong resistance to both drying and swelling

*Md. Tariful Islam Mredha, Hong Hieu Le, Jiaxi Cui, and Insu Jeon **

Materials: Acrylamide (AAm), *N,N′*-methylenebisacrylamide (MBAA), 2,2-azobisisobutyronitrile (AIBN), benzene, calcium chloride ($CaCl_2$), and sodium chloride (NaCl) were received from Daejung Chemicals & Metals Co., Ltd., Korea. Ethyl acetate, n-hexane and acetone were received from Samchun Pure Chemical Co., Ltd., Korea. Ammonium persulfate (APS) and *N,N,N′,N′*-tetramethylethylenediamine (TEMED) were purchased from Sigma-Aldrich Co., USA. Hydrophobic monomers—octyl acrylate (OA), dodecyl acrylate (DA), and stearyl acrylate (SA)—were received from Tokyo Chemical Industrial (TCI) Co., Ltd., Japan. Silicone oil (100, 1000, and 2000 cSt at 25 °C) and castor oil were purchased from Saehan Silichem Co., Ltd., Korea and USDA Organic, Korea, respectively. Na-alginate was received from Junsei Chemical Co., Ltd., Japan. All chemicals were used as received, without further purification. Seawater was collected from the South Sea of Korea. Ultrapure deionized water was used in all cases, unless otherwise noted.

Fabrication of polyacrylamide (PAAm) hydrogel: A precursor solution of 3 M AAm monomer, 0.1 mol% MBAA (with respect to the monomer concentration), and 0.1 mol% APS (with respect to the monomer concentration) was prepared in deionized water. The solution was purged with nitrogen

gas to remove any dissolved oxygen. Then, 2 µL/mL TMED was added to the solution, and the mixture was transferred to a predesigned glass mold (prepared by sandwiching two glass plates separated by a 3 or 2 mm thick silicone rubber sheet) for polymerization. After 12 h of incubation at room temperature (~25 °C), the obtained PAAm hydrogel was removed from the mold and used for coating and other characterizations.

Fabrication of Ca-alginate/PAAm hydrogel: A Ca-alginate/PAAm tough double-network hydrogel was prepared via a two-step method based on a previously reported procedure.[S1] First, a precursor solution of 1.56 wt% Na-alginate and 1.75 M AAm monomer (AAm/Na-alginate weight ratio = 8:1) together with 0.03, 0.03, and 0.15 mol% (relative to the AAm concentration) of MBAA, APS, and TMED, respectively, was prepared in deionized water. The solution was then transferred to a predesigned glass mold (prepared by sandwiching two glass plates separated by a 3 or 2 mm thick silicone rubber sheet) and polymerized at 50 °C for 4 h. In the second step, the as-prepared Na-alginate/PAAm hydrogel was removed from the glass mold and immersed in a 0.5 M aq. $CaCl_2$ solution for 4 h for ionic cross-linking. Thus, we obtained the tough Ca-alginate/PAAm double-network hydrogel, which was used for coating and other characterizations.

Fabrication of double-hydrophobic-coated hydrogels: Double-hydrophobic-coating through a quenching process was employed to prepare hydrogels with strong resistance to drying and swelling. First, we applied the method to a PAAm hydrogel. A PAAm hydrogel of the desired shape was immersed in a 4 wt% AIBN/benzene solution (where AIBN served as the thermal initiator for the polymerization) for 4 h at room temperature (~25 °C) and 20 min at 120 °C. Because AIBN/benzene is immiscible with water, it was adsorbed only on the surface layer of the hydrogel, by replacing the relatively unstable water on the outermost layer of the hydrogel. Then, the hydrogel was dried at 120 °C for a short period (~2 min), causing evaporation of the low boiling point benzene, which left

only AIBN trapped on the surface layer of the hydrogel. Subsequently, the hydrogel was immersed in a preheated (120 °C) liquid hydrophobic monomer (OA, DA, SA, or DA/SA (1:1 by volume)) mixture for polymerization. At this high temperature, the hydrophobic monomer penetrated the surface layer of the hydrogel and was polymerized by the pre-trapped AIBN present there. After ~15, ~30, or ~45 min of polymerization, the hydrophobic polymer-coated hydrogel was removed from the monomer solution and immersed in a preheated (120 °C) silicone oil bath for 15 min. The hot oil easily penetrated the hydrophobic polymer layer of the hydrogel surface. It also washed away any unreacted monomer present there. Then, the obtained double-hydrophobic-coated (hydrophobic polymer and oil) hydrogel was removed from the hot oil and quenched quickly via immersion in a silicone oil bath kept at room temperature (~25 °C) for 15 min. Next, the gel was removed from the oil bath, and excessive oil was wiped from the surface. This double-hydrophobic-coated hydrogel was used for further characterization. For comparison, coatings were applied at different silicone oil bath temperatures (120, 80, and 40 °C) followed by subsequent quenching at room temperature (25 °C). To demonstrate the versatility of the proposed method, the coating was also performed on a Ca-alginate/PAAm double-network hydrogel using the same procedure. Additionally, castor oil was used instead of silicone oil to demonstrate the broad applicability of the method. The air-drying and water-swelling properties of the gels were evaluated by exposing the disk-shaped gels (diameter = 10 mm, thickness = 3 mm) to air and water environments and checking their weight at regular intervals over 7 d. The coated hydrogels were first kept in air for 7 d and then immersed in water for 7 d to evaluate their swelling properties. Air-drying behavior was evaluated under ambient conditions (temperature: ~25 °C; humidity: 50%–60%), and water-swelling behavior was evaluated in pure water (~25 °C), seawater (~25 °C), and a physiological saline solution (0.16 M aq. NaCl at 37 °C). At least 3 samples were tested to evaluate the air-drying and swelling properties and the data are presented as mean values with mean absolute deviations.

Determination of coating density: Three double-hydrophobic-coated gels (diameter: ~10 mm, thickness: ~3 mm) were immersed into ~50 mL hexane and stirred overnight. Subsequently, the gels were removed and the extract was dried. The process extracted the entire hydrophobic component from the gel surface, which was confirmed by water-contact-angle measurement on the gel surface (Figure S13). The dried extract (hydrophobic polymer and silicone oil) was weighted and the coating density was determined using Equation 1.

Coating density ($g \cdot cm^{-2}$) = Weight of hydrophobic polymer and oil (g)/Surface area of gel (cm^2) (1)

Determination of oil content in double-hydrophobic-coated layer: Three double-hydrophobic-coated gels (diameter: ~10 mm, thickness: ~3 mm) were immersed into ~50 mL ethyl acetate and kept overnight. After that the gels were removed, dried, and washed with acetone. The weight of the collected dried extract indicates the amount of silicone oil absorbed by the coating, which was further confirmed by thermogravimetric analyses (TGA) (Figure S14).

Mechanical characterizations: All mechanical tests—tensile, compression, and adhesion tests—were performed using a commercial tensile test machine (model TO-100-1C, TESTONE Co. Ltd., Korea). All tests were conducted under ambient conditions (~25 °C). A 10 kgf load cell was used for the tensile and adhesion tests, and a 100 kgf load cell was used for the compression tests. At least three tests were performed for each sample and the data are presented as mean values with mean absolute deviations.

Tensile tests. Rectangular samples (length × width × thickness = 30 × 5 × 2 mm^3) were used for the tensile test. Both ends of the length direction were clamped in the tensile tester, and the ~10

mm initial distance between the clamps was maintained. The upper clamp was connected to a load cell. The tensile test was performed by moving the upper clamp upward until fracture, with a deformation velocity of 500%·min^{-1}.

Compression tests. Compression tests were performed using disk-shaped samples (diameter = 10 mm, thickness = 3 mm). Each sample was placed between two parallel steel plates, and the upper plate was connected to a load cell. Compression was performed by moving the upper plate downward with a deformation velocity of 100%·min^{-1}. Cyclic compression tests were also performed, using the same experimental setup.

Adhesion tests. The adhesion properties were evaluated via a tack test. Disk-shaped samples (diameter = 10 mm, thickness = 3 mm) were used. The setup included two parallel plates consisting of one upper surface (indenter), which was connected to a load cell, and one bottom surface, to which a glass plate (adhesion substrate) was fixed. The upper surface of the sample was first glued to the indenter. The indenter was moved down until the gel attached to the glass plate (bottom surface) with a compression load of 1 N. The load was maintained for 1 min, and then the indenter was moved upward to release the load. The tack test was then performed immediately by moving the indenter upward with a deformation velocity of 100%·min^{-1} until the gel completely detached from the glass substrate. The adhesive force as a function of displacement was obtained as a curve, which was used to calculate the adhesive strength and energy.

Structural observations: We observed the structures of the materials using scanning electron microscopy (SEM; S-4700, Hitachi, Japan). The samples were first air-dried after cutting through the cross-section, which exposed the bulk region (non-coated part) to facilitate the drying process. Then, the dried samples were coated with platinum using an ion-sputtering system and observed via SEM, from both the surface and cross-sectional directions.

Contact angle measurements: The contact angle was measured by dropping 5 µm of water from a pipette tip onto the gel surface. A digital photograph was taken immediately from the side view (parallel to the sample surface plane), which was used for calculating the contact angle.

Supporting Figures

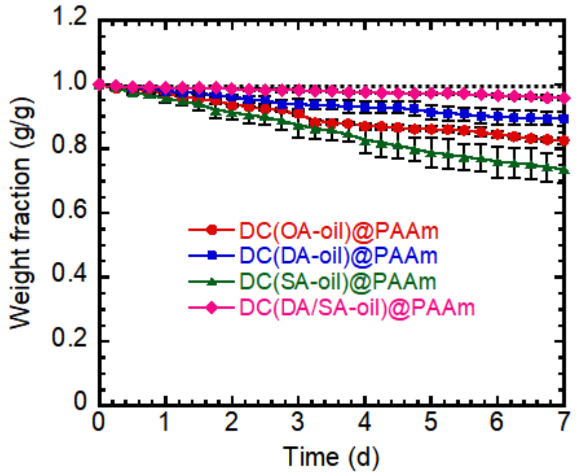

Figure S1. Comparison of the air-drying properties of double-coated hydrogels fabricated using different hydrophobic polymers (OA, DA, SA, and DA/SA = 1:1).

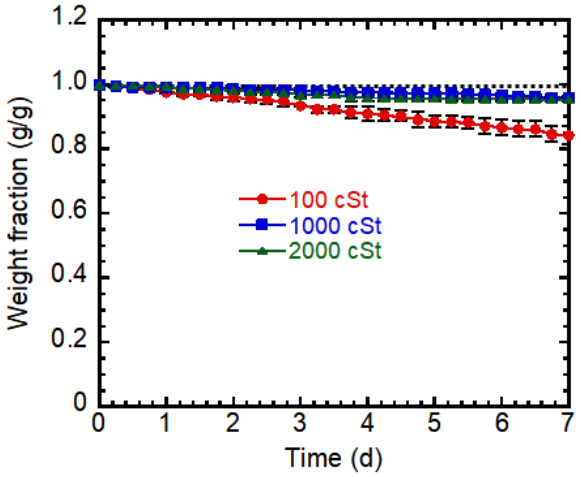

Figure S2. Air-drying properties of the DC(DA/SA-oil)@PAAm hydrogel fabricated using silicone oil of different viscosities.

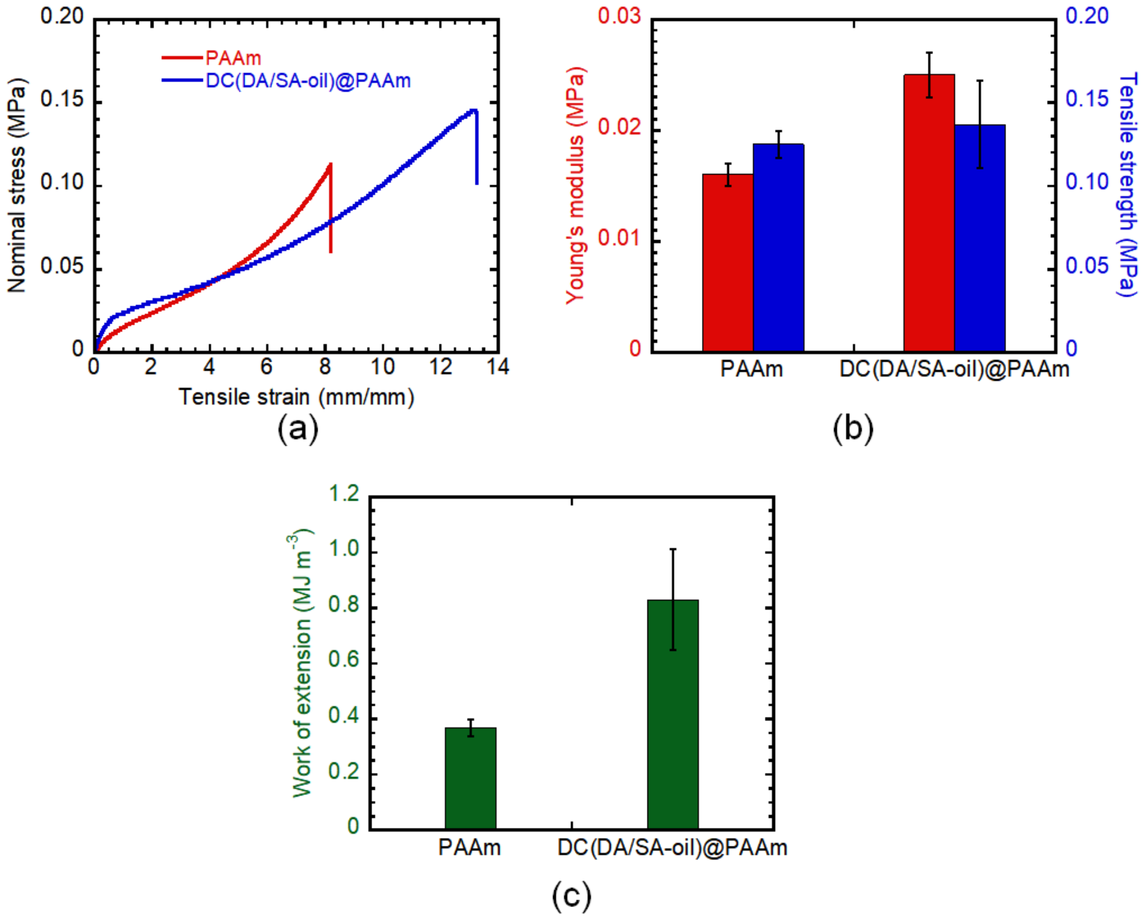

Figure S3. Tensile properties of PAAm and DC(DA/SA-oil)@PAAm hydrogels. (a) Tensile stress–strain curves, (b) Young's modulus and tensile strength, and (c) work of extension.

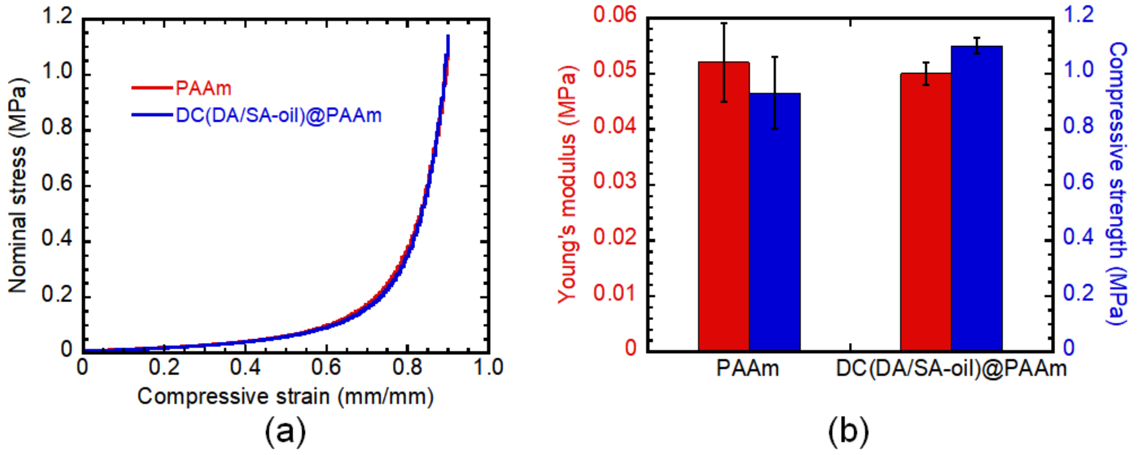

Figure S4. Compression properties of PAAm and DC(DA/SA-oil)@PAAm hydrogels. (a) Compressive stress–strain curves and (b) Young's modulus and compressive strength (at a compressive strain of 0.9).

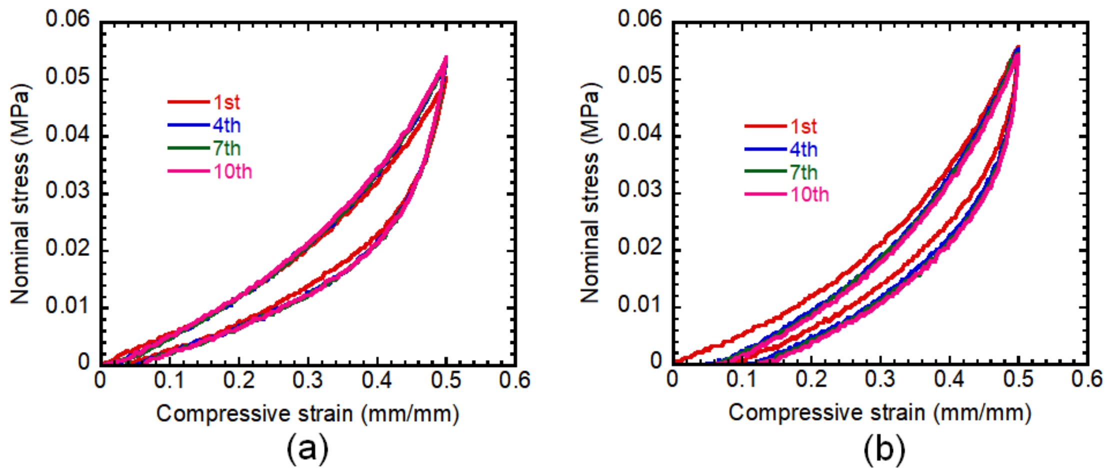

Figure S5. Ten consecutive loading–unloading cycles of (a) PAAm and (b) DC(DA/SA-oil)@PAAm hydrogels up to a compressive strain of 0.5. For simplicity, only the 1st, 4th, 7th, and 10th cycles are shown.

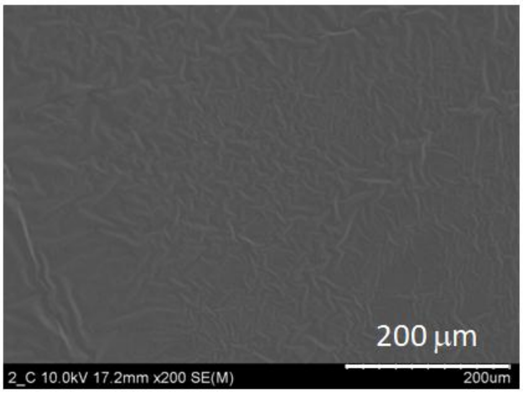

Figure S6. SEM image of the surface of the PAAm hydrogel.

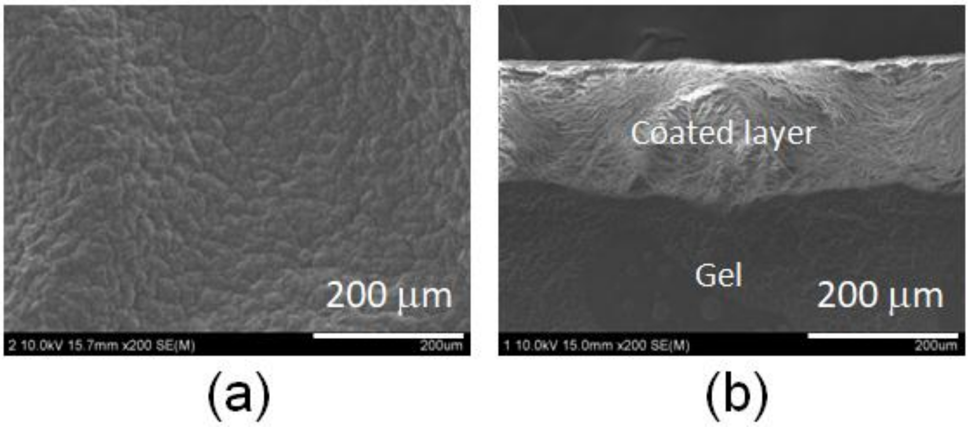

Figure S7. SEM images of the SC(DA/SA)@PAAm hydrogel showing (a) surface and (b) cross-sectional views.

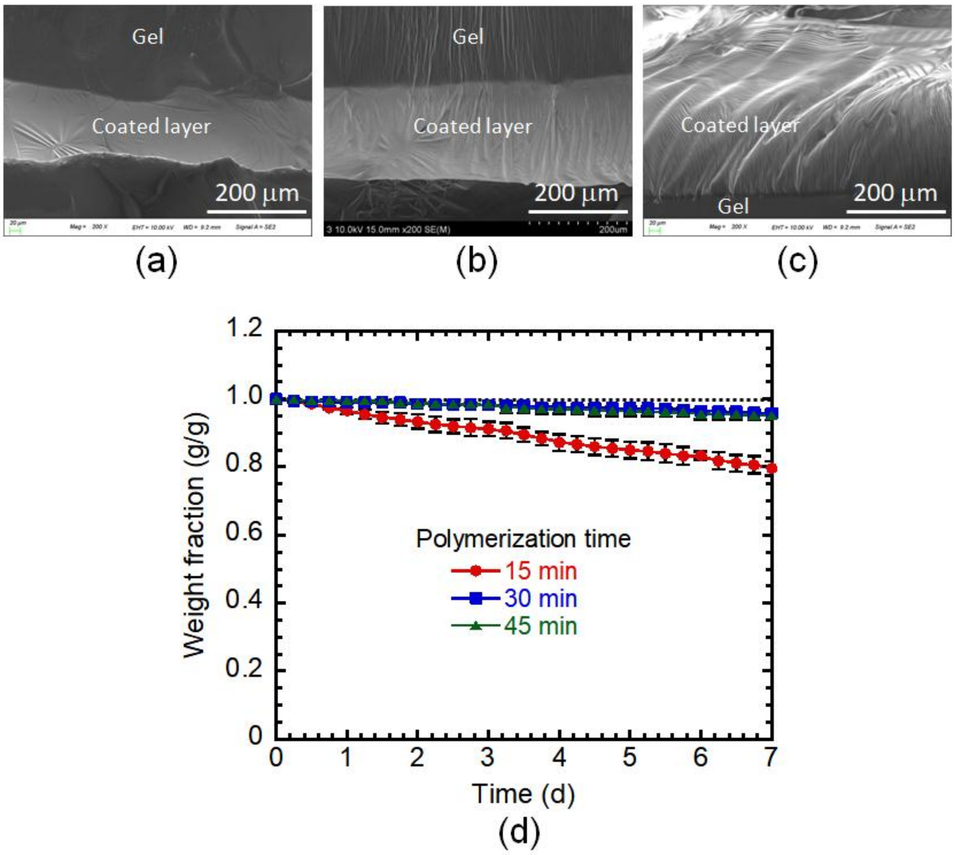

Figure S8. Cross-sectional SEM images of the DC(DA/SA-oil)@PAAm hydrogel obtained by polymerization of the hydrophobic monomers for (a) 15-min, (b) 30-min, and (c) 45-min. (d) Air-drying performance of these hydrogels.

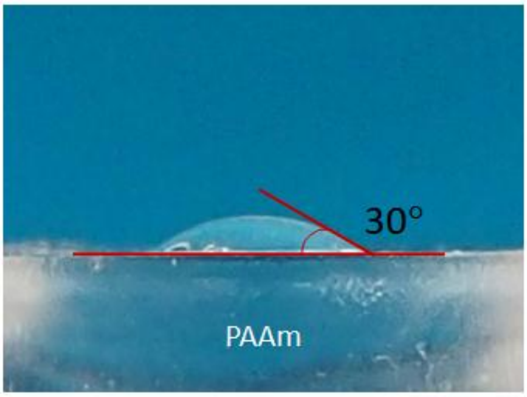

Figure S9. Measurement of the contact angle between the water droplet and the PAAm hydrogel surface.

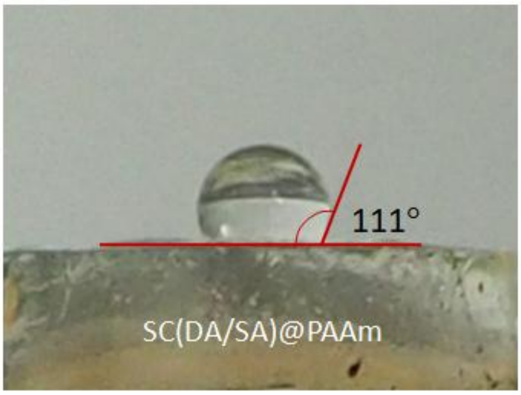

Figure S10. Measurement of the contact angle between the water droplet and the SC(DA/SA)@PAAm hydrogel surface.

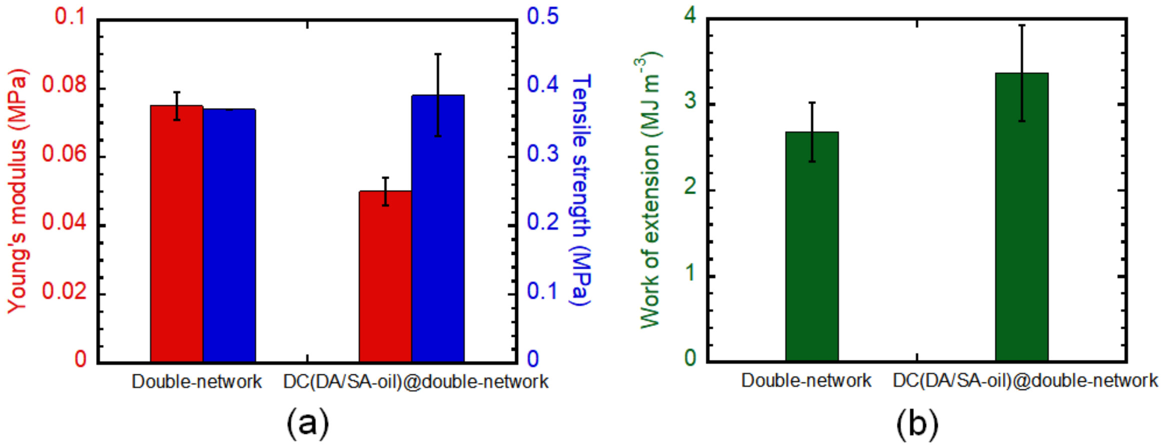

Figure S11. Tensile properties of double-network and DC(DA/SA-oil)@double-network hydrogels. (a) Young's modulus and tensile strength and (b) work of extension.

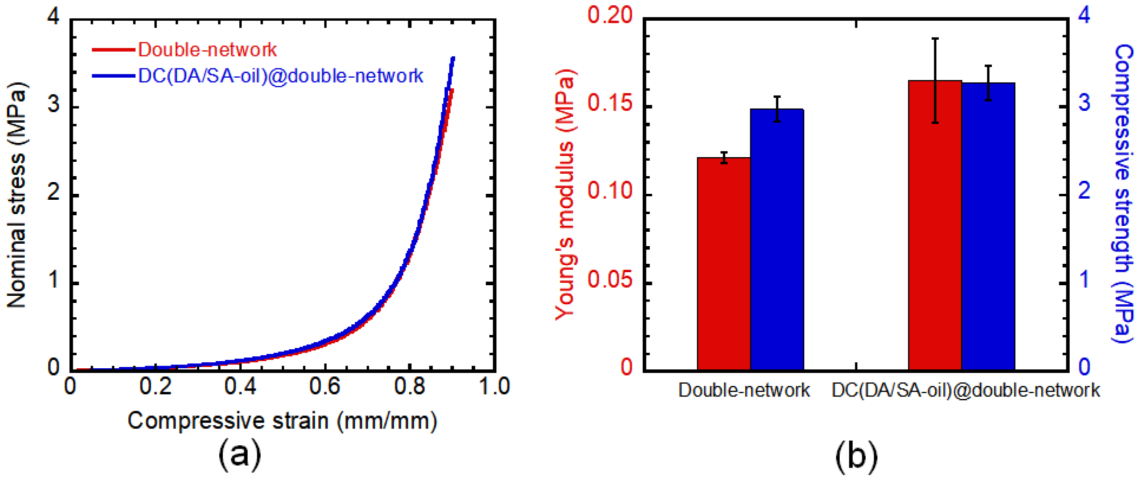

Figure S12. Compression properties of double-network and DC(DA/SA-oil)@double-network hydrogels. (a) Compressive stress–strain curves and (b) Young's modulus and compressive strength (at a compressive strain of 0.9).

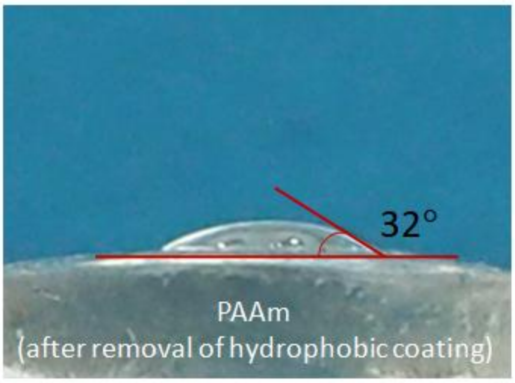

Figure S13. Water-contact-angle on PAAm hydrogel surface after extraction of the coated layer (hydrophobic polymer and oil). The measurement is quite similar to that of the fresh PAAm hydrogel (Figure S9).

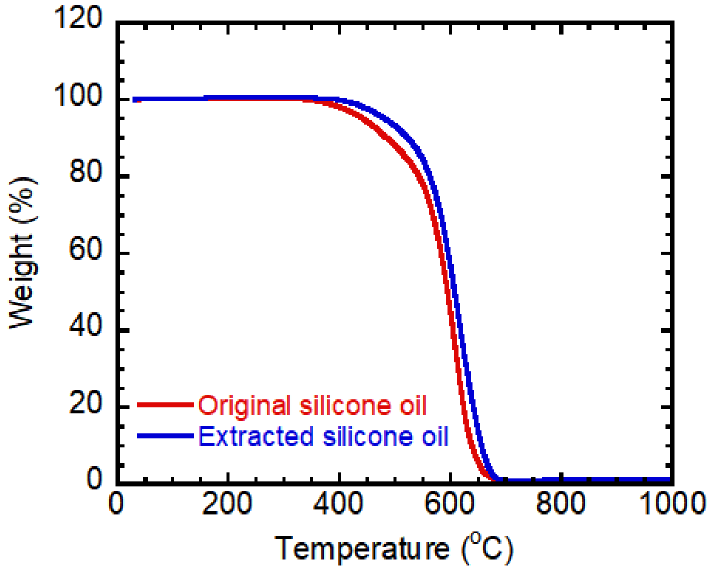

Figure S14. TG behavior of silicone oil extracted from DC(DA/SA-oil)@PAAm gel. The behavior is quite similar to that of the original silicone oil. Measurement was conducted by TGA (TGA2, METTLER TOLEDO, SWISS) from 30 to 1000 °C at a heating rate of 10 °C/min under a nitrogen atmosphere.

Supporting Table

Table S1. Comparison of the adhesive strength of various reported hydrogel adhesives with those of our hydrogel.

Materials	Substrate	Adhesive strength (kPa)	Measurement method
PEG-DA/PEG-PBA[S2]	Glass	~4	Lap-shear test
PVA/FSWCNT/PDA[S3]	Glass	9.2	Lap-shear test
PDA/PAAm[S4]	Porcine skin	6–15.2	Lap-shear test
Polyampholyte hydrogel[S5]	Glass	2–13	Tack test
Polyelectrolyte hydrogel[S6]	Glass	~7.8	Lap-shear test
Nanoparticle solution[S7]	Hydrogel, tissue	1.5–6	Lap-shear test
This study	Glass	6.4 ± 0.4	Tack test

References

[S1] C. H. Yang, M. X. Wang, H. Haider, J. H. Yang, J.-Y. Sun, Y. M. Chen, J. Zhou, Z. Suo, *ACS Appl. Mater. Interfaces* **2013**, *5*, 10418.

[S2] M. Shan, C. Gong, B. Li, G. Wu, *Polym. Chem.* **2017**, *8*, 2997.

[S3] M. Liao, P. Wan, J. Wen, M. Gong, X. Wu, Y. Wang, R. Shi, L. Zhang, *Adv. Funct. Mater.* **2017**, *27*, 1703852.

[S4] L. Han, L. Yan, K. Wang, L. Fang, H. Zhang, Y. Tang, Y. Ding, L.-T. Weng, J. Xu, J. Weng, Y. Liu, F. Ren, X. Lu, *NPG Asia Mater.* **2017**, *9*, e372.

[S5] P. Rao, T. L. Sun, L. Chen, R. Takahashi, G. Shinohara, H. Guo, D. R. King, T. Kurokawa, J. P. Gong, *Adv. Mater.* **2018**, *30*, 1801884.

[S6] W. Li, R. Feng, R. Wang, D. Li, W. Jiang, H. Liu, Z. Guo, M. J. Serpe, L. Hu, *J. Mater. Chem. B* **2018**, *6*, 4799.

[S7] S. Rose, A. Prevoteau, P. Elzière, D. Hourdet, A. Marcellan, L. Leibler, *Nature* **2014**, *505*, 382.
